# Automated generation of consistent models using qualitative abstractions and exploration strategies

**DOI:** 10.1007/s10270-021-00918-6

**Published:** 2021-09-17

**Authors:** Aren A. Babikian, Oszkár Semeráth, Anqi Li, Kristóf Marussy, Dániel Varró

**Affiliations:** 1 Department of Electrical and Computer Engineering, McGill University, 3480 Rue University, Montréal, QC H3A 0E9 Canada; 2 Department of Measurement and Information Systems, Budapest University of Technology and Economics, Magyar tudósok krt. 2, Budapest, 1117 Hungary; 3 Department of Computer Science, ETH Zürich, Rämistrasse 101, Zürich, 8092 Switzerland

**Keywords:** Model generation, Partial model, Graph solver, SMT-solver, Numeric solver, Exploration strategy, Test scenario synthesis

## Abstract

Automatically synthesizing consistent models is a key prerequisite for many testing scenarios in autonomous driving to ensure a designated coverage of critical corner cases. An inconsistent model is irrelevant as a test case (e.g., false positive); thus, each synthetic model needs to simultaneously satisfy various structural and attribute constraints, which includes complex geometric constraints for traffic scenarios. While different logic solvers or dedicated graph solvers have recently been developed, they fail to handle either structural or attribute constraints in a scalable way. In the current paper, we combine a structural graph solver that uses partial models with an SMT-solver and a quadratic solver to automatically derive models which simultaneously fulfill structural and numeric constraints, while key theoretical properties of model generation like completeness or diversity are still ensured. This necessitates a sophisticated bidirectional interaction between different solvers which carry out consistency checks, decision, unit propagation, concretization steps. Additionally, we introduce custom exploration strategies to speed up model generation. We evaluate the scalability and diversity of our approach, as well as the influence of customizations, in the context of four complex case studies.

## Introduction

**Motivation.** The recent increase in popularity of cyber-physical systems (CPSs) such as autonomous vehicles has resulted in a rising interest in their safety assurance. Since existing tools and approaches commonly represent CPSs as (typed and attributed) graph models [43], automated generation of test models has become a core challenge for their effective testing. Recent testing approaches [9] use simulators to place the CPS under test in challenging traffic scenarios defined by (generated) test configurations. In such approaches, the CPS is considered as a black box and its safety is evaluated at the system level, without direct handling of internal components and their interactions. In order to synthesize adequate (realistic) test data for safety assurance of CPSs, data generation approaches must handle complex structural and numeric constraints.

**Problem statement.** Unfortunately, the automated synthesis of consistent graph-based models that satisfy (or deliberately violate) a set of well-formedness constraints is a very challenging task. While various underlying logic solvers like SAT, SMT (Satisfiability Modulo Theories) or CSP (Constraint Satisfaction Problem) solvers have been repeatedly used for such purposes in tools, like in USE [26, 27], UML2CSP [15], Formula [38], various theorem provers [5], or Alloy [35] thanks to many favorable theoretical properties (e.g., soundness or completeness) such solvers primarily excel in detecting inconsistencies and not in deriving models used as test cases. Rather than being used to address the model generation task as a whole, such specialized solvers (e.g., dReal [23]) may be more useful for handling only specific aspects (e.g., numeric constraints) of model generation. In fact, although there does exist research [4, 75, 76] that uses such solvers for testing purposes, the use of such solvers as a stand-alone model generation tools is frequently hindered by the lack of scalability [71, 82] (i.e., the size of generated models is limited) and diversity [37, 70] (i.e., generated models often have similar or identical structure).

Recent model generators [71, 81, 82] have successfully improved on scalability by lifting the model synthesis problem on the level of graph models by using meta-heuristic search [81], possibly within a hybrid approach alongside an SMT-solver [82]. Alternatively, partial model refinement [71] can be used as search strategy, while efficient query/constraint evaluation engines [83, 86] validate the constraints during state space exploration. However, there are also important restrictions imposed by these tools such as lack of completeness [81, 82] or lack of attribute handling [71] in constraints.

**Contributions.** In this paper, we propose a model generation technique which can automatically derive consistent graph models that satisfy both structural and attribute constraints. For that purpose, the structural constraints are satisfied along partial model refinement [71], while attribute constraints are satisfied by repeatedly calling the Z3 SMT-solver [17] or the dReal quadratic solver (like [82]). We define refinement units (in analogy with an abstract DPLL procedure modulo theories (Davis–Putnam–Logemann–Loveland) [51] or SMT-solvers [50]) with consistency checking, decision, unit propagation and concretization steps to enable a bidirectional interaction between a graph solver and a numeric solver where a decision in one solver can be propagated to the other solver and vice versa.

Specific contributions of the paper include:

- **Precise semantics:** We define 3-valued logic semantics for evaluating structural and attribute constraints over partial models.
- **Qualitative abstractions:** We propose qualitative abstractions to uniformly represent attribute constraints as (structural) relations in a model.
- **Mapping for numeric constraints:** We define a mapping from attribute constraints to a numerical problem interpreted by a numeric solver.
- **Model generation approach:** We propose a generic model generation strategy with bidirectional interaction between a structural solver and two numeric solvers to handle *int* or *double* constraints.
- **Custom exploration strategy:** We propose a technique to define custom explorations for model generation to exploit domain-specific hints.
- **Evaluation:** We evaluate a prototype implementation of the approach on four case studies to assess scalability and diversity properties of model generation, as well as the influence of customizations.

This paper extends our earlier work in [67] by

1. introducing a *new motivating example* for traffic scenario generation with nonlinear constraints;
2. integrating an *approximate numeric solver* dReal [23] to handle non-linear geometric constraints;
3. providing a *detailed mapping* from attribute constraints to numerical problems;
4. introducing *custom exploration strategies*;
5. extending *experimental evaluation* with a complex case study (traffic scenario) and research question.

**Added value.** Our approach provides good scalability for automatically generating consistent models with structural and attribute constraints while still providing completeness and diversity. We also successfully generate models of traffic scenarios operating in physical space, which is a promising result toward complex simulation analysis for safety assurance of CPSs.

**Structure of the paper.** The rest of the paper is structured as follows: Sect. 2 describes the motivating case study related to traffic scenario generation. Section 3 presents a running example and summarizes core concepts pertaining to partial models and their refinements. Sections 4 and 5 detail our model generation approach that combines structural and numeric reasoning. Section 6 provides evaluation results of our proposed approach for four case studies. Section 7 overviews related approaches available in the literature. Finally, Sect. 8 concludes the paper.

## Motivating example

### The Crossing Scenario domain

**Fig. 1:**
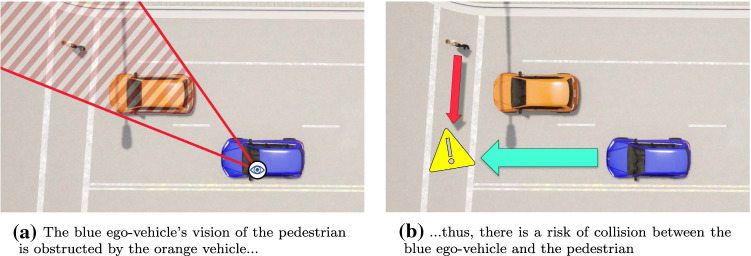
Traffic scenario that involves an area with limited visibility

We illustrate various challenges of model generation and state space exploration in the context of critical traffic scenarios for autonomous driving. Specifically, we aim at generating instances of traffic scenarios where the vision of the vehicle-under-test (referred to as the ego-vehicle [25, 60]) is obstructed by the presence of other actors. Such scenarios have been identified as key challenges for the development of autonomous vehicle safety^1^. A sample scenario is shown in Fig. 1, where the actors are placed in such a way that the ego-vehicle (blue) is unable to see the pedestrian that is crossing the road due to the presence of the orange vehicle. As a result, there is a risk of collision between the ego-vehicle and the pedestrian if both of these actors decide to cross the intersection simultaneously.

By using the novel model generation techniques proposed in this paper, we aim to automatically synthesize traffic scenarios where actors are positioned in such a way that the ego-vehicle and the target pedestrian cannot see each other initially. Furthermore, these actors are given velocities such that they will collide with each other within a certain time limit to enforce reaction. These requirements correspond to complex geometric (numeric) constraints that must be handled during model generation.

To precisely capture this modeling domain, we use a metamodel (Sect. 2.2) and complex well-formedness (WF) constraints defined by graph patterns (in the VQL language [85, 86]) in Sect. 2.3.

**Fig. 2:**
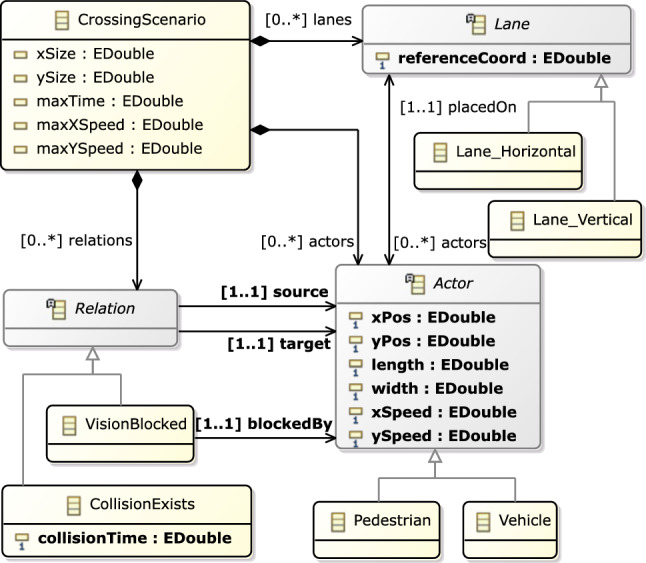
Metamodel of the Crossing Scenario domain

### Metamodel

A  is composed s, s and s between actors. It also contains numerical attribute that act as bounds for actor positions ( and ), actor speeds ( and ) and collision time () between actors.

 objects are static elements in the scenario which are provided as input for model generation. In our metamodel, we include abstractions for vertical and horizontal s, which refers to their orientation when seen from a bird’s-eye view (see Fig. 12e for an example). This ensures that the respective lanes in a scenario intersect with each other. Each  contains a  attribute which designates the left boundary for vertical lanes, and the bottom boundary for the horizontal lanes. Lanes have a predefined width, which is identical for all lanes.

*Scenario specifications*, such as the existence of certain s or certain s between actors, are also included as inputs for model generation. For example, in the scenario shown in Fig. 1b, model generation inputs would enforce the existence of two actors (the ego-vehicle and the target pedestrian) which have their vision blocked and which will eventually collide (if there is no change in their behavior). It is the task of the model generator to create a new actor, place all actors appropriately and set their speeds such that the scenario specifications are satisfied.

Actors are placed on a lane and have a concrete numeric position (, ), size (, ) and speed (, ).

 objects defined over two actors ( and ) represent qualitative abstractions of certain sequences of events or trajectories. For the sake of brevity, we only include two different types of s, namely  and . Relation  signifies that the  and  actors are unable to see each other because their line of sight (vision) is blocked by another actor which is physically placed between them. Relation  denotes that the two actors involved are given velocities such that they will collide at a time . The metamodel can be extended to incorporate further  types.

Such qualitative abstractions help enforce complex trajectories and behaviors without the need for continuously handling the exact attribute values. For example, if a relation (A1, A2) exists between actors A1 and A2, then the numerical attributes of those actors are set up in a way that they would surely collide along the default behavior (without further control intervention like braking), but not vice versa.

### Well-formedness constraints

Our motivating example includes 32 constraints to restrict various parts of the metamodel. Actors have bounded positions, sizes and speeds, which are further constrained by the orientation and  of the lane on which they are placed. A minimum Euclidean distance is also enforced between any pair of actor to avoid overlaps. Qualitative relations (see Sect. 2.2) enforce custom constraints on attribute values.

**Fig. 3:**
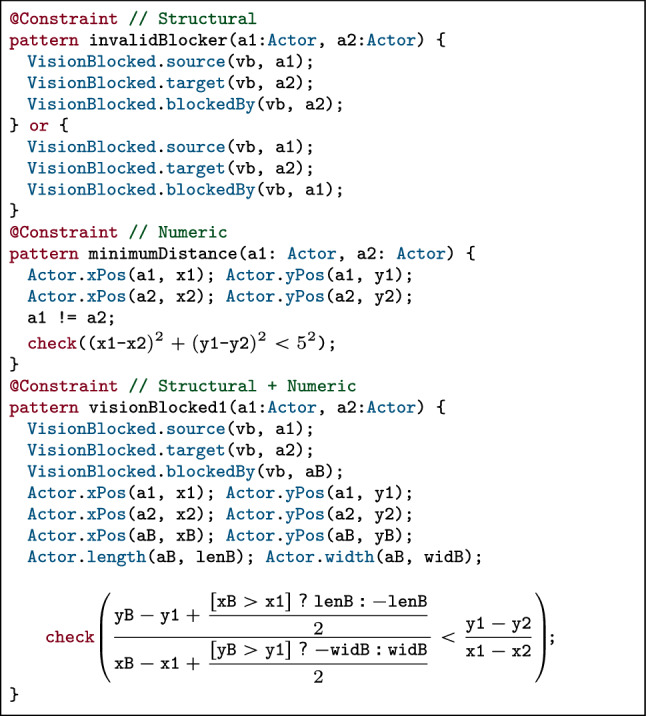
Representative structural & numeric constraints from the Crossing Scenario domain

This case study includes complex geometric constraints, quadratic inequalities, non-constant divisions and numeric *if-then-else* blocks. The *violating cases* of three representative constraints, implemented as VQL graph patterns ([85, 86]), are shown in Fig. 3.

: checks if any actor in a  relation is not the blocking actor;
: checks (using slopes) that the blocking actor of a  relation is physically placed between the  and  actors;
: enforces a minimum Euclidean distance of 5 between any pair of distinct actors.

In this paper, we use color coding to separate  and  reasoning. The first constraint is a  (i.e., only navigation along object references). The second constraint is a  which accesses the  and  attributes of actors a1 and a2 to check the Euclidean distance between them. The third constraint contains both  and  which mutually depend on each other. (A) If the vision between a1 and a2 is blocked by a new actor aB, then a new numeric constraint needs to be enforced between their positions and sizes ($\rightarrow$  dependency). (B) If the positions and sizes of actors a1, a2 and aB are already determined, then a new  reference pointing to one of the actors may (or must not) be added ($\rightarrow$  dependency).

The Crossing Scenario domain extends [67] by showcasing complex, nonlinear numeric constraints over real numbers. These constraints can only be handled efficiently by specialized numeric solvers, such as dReal [23], which are limited in scalability and diversity when reasoning over structural constraints.

Consistent model generation is further complicated by the existence of mutual dependencies between structural and numeric constraints. Thus, generating models that conform to the Crossing Scenario domain requires an intelligent integration and bidirectional interaction between underlying numeric and structural (graph) solvers. In the paper, the bidirectional interaction is exemplified for numerical attributes, but the conceptual framework is applicable to attributes of other domains (e.g., strings, bitvectors) assuming the existence of an underlying solver (e.g., SMT-solver) for the background theory of the respective attribute.

## Preliminaries

### Running example

**Fig. 4:**
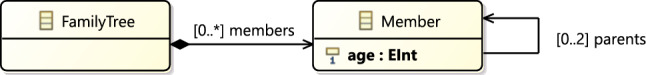
Metamodel of the Family Tree domain

**Fig. 5:**
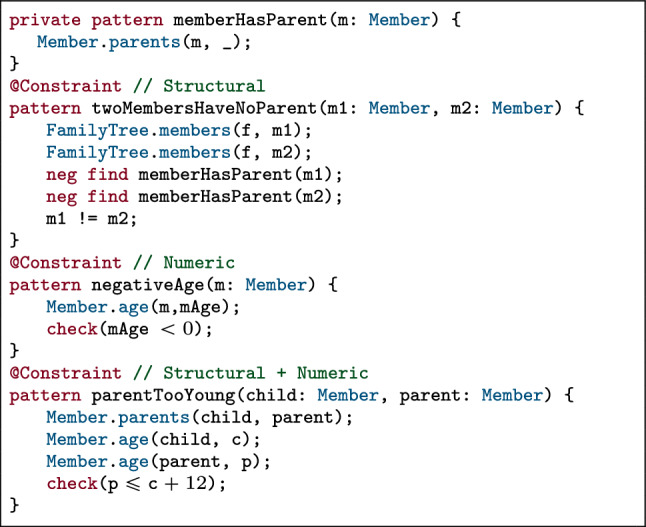
Structural & numeric constraints in the Family Tree domain

To succinctly present the formal background of our model generation approach, we present a simple running example of family trees with a metamodel (Fig. 4) and WF constraints defined by graph patterns (Fig. 5). This domain is intentionally chosen to contain only few concepts, while it can demonstrate all key technical challenges of constraint evaluation.

A  contains s with an integer  attribute.  are related to each other by  relations. The *violating cases* of the three WF constraints are defined by VQL graph patterns that all consistent family tree models need to respect:

: There is at most one member in a family tree without a parent;
: All age attributes of family members are non-negative numbers;
: There must be more than 12 years of difference between the  of a parent and a child.

### Domain-specific partial models

*Domain specification* We formalize the concepts in a target domain $\langle \Sigma ,\alpha \rangle$ using an algebraic representation with signature $\Sigma$ and arity function $\alpha :\Sigma \rightarrow {\mathbb {N}}$. Such a signature  can be easily derived from EMF-like formalisms [84].

- Unary predicate symbols  () are defined for each *EClass* and *EEnum* in the domain,  denotes the *EBoolean* type,  denotes integer numbers types like *EInt* or *EShort*, etc.
- Binary predicate symbols  ( ) are defined for each *EReference* and *EAttribute* in the metamodel. For example,  represent the *parent* reference between two *Members*, and  represents the *age* attribute relation between a *Member* and an *EInt*.
- Structural predicate symbols  are *n*-ary predicates derived from graph queries (, the number of formal parameters of a graph query); e.g.,  is a binary predicate symbol.
- Attribute predicate symbols  represent *n*-ary predicates derived from attribute (*check*) expressions of queries (); e.g.,  is a binary attribute predicate with parameters *c* and *p*.
- The unary symbol  denotes the *existence* of objects.
- The binary symbol  explicitly represents the *equivalence* relation between two objects.

*Partial models* Partial models can explicitly represent uncertainty in models [18, 64], which is particularly relevant for intermediate steps of a model generation process. We use 3-valued partial models where the traditional  () and  () are extended with a third truth value  to denote  structural parts of the model [29, 61, 72]. Similarly, we extend the domain of traditional  (e.g.,  or ) with  to denote an .

#### Definition 1

(Numerical partial model) For a signature $\langle \Sigma ,\alpha \rangle$, a *numerical partial model* is a logic structure  where:

is the finite set of objects in the model,
gives a 3-valued logic interpretation for each symbol  as ,
gives a numeric value interpretation for each object in the model: .

Note that this definition uniformly handles domain objects (e.g., ) and data objects (e.g., ), which is frequently the case in object-oriented languages. Next, we capture some regularity restrictions to exclude irrelevant (irregular) partial models:

#### Definition 2

(Regular partial models) A partial model  is *regular*, if it satisfies the following conditions:

(non-existing objects are omitted)
( is reflexive)
( is symmetric)
(two different objects cannot be equivalent)
(domain objects have no values)
(objects with values are numbers)
(only natural numbers are bound to  objects)

#### Example 1

Figure 9 illustrates partial models. In **State 1**, we have three concrete objects (where  and  are ): *f*1 and a  *m*1, and an unbound  data object *a*1 (with  value). The partial model also contains an abstract *“new objects”* node that represents multiple potential new nodes (using  values for  and dashed borders for ), and a *“new integers”* node representing the potential new integers. In Fig. 9, predicates with value  are denoted by solid lines (as for the  edge between *f*1 and *m*1 in **State 1**) and predicates with value  are denoted by dashed lines (like the potential  edge in **State 1**).

### Refinement and concretization

During model generation, the level of uncertainty in partial models will be gradually reduced by refinements. In a refinement step, uncertain  values can be refined to either  or , or unbound values  are refined to concrete numeric values. This is captured by an information ordering relation  where an  is either refined to another value *Y*, or $X=Y$ remains equal. An information ordering can be defined between numeric values *x* and *y* similarly 

A refinement from partial model *P* to partial model *Q* is a mapping that respects both information ordering relations ().

#### Definition 3

(Partial model refinements) A refinement  from regular partial model *P* to regular partial model *Q* is defined by a refinement function between the objects of the partial model  which respects information ordering:

- For each *n*-ary symbol $s\in \Sigma$, each object , and for each refinement $q_1\in ref (p_1),\ldots ,q_n\in ref (p_n)$:
- For each object $p\in {\mathcal {O}}_{P}$ and its refinement $q\in ref (p)$:
- All objects in *Q* are refined from an object in *P*, and existing objects  must have a non-empty refinement.

Model generation along refinements eventually resolves all uncertainties to obtain a concrete model.

#### Definition 4

(Concrete partial model) A regular (see Definition 2) partial model *P* is *concrete*, if (a) ${\mathcal {I}}_P$ does not contain  values, and (b) ${\mathcal {V}}_P$ does not contain  values for integer and real data objects (for object *o* where  or ).

#### Example 2

Figure 9 illustrates several refinement steps. Between **State 0** and **State 1**, *new object* is split into two objects by refining  to  between *new object* and *m*1, creating one concrete object *m*1 by refining  on *m*1 to . Moreover, type  is refined to ,  refined to , and reference  from *f*1 to *m*1 is refined to . Eventually, the value of data object *a*1 is refined from  to  in **State 4.2**.

### Constraints over partial models

*Syntax* Both structural (logical) and numeric constraints can be evaluated on partial models. For each graph pattern we derive a *logic predicate* (LP) defined as , where $\varphi$ is a *logic expression* (LE) constructed inductively from the pattern body as follows (assuming the standard precedence for operators).

- if $s \in \Sigma$ is an n-ary predicate symbol (i.e., , , , ,  or ) then $s(v_1,\ldots ,v_n)$ is a logic expression;
- if $\varphi _1$ and $\varphi _2$ are logic expressions, then $\varphi _1\vee \varphi _2$, $\varphi _1\wedge \varphi _2$, and $\lnot \varphi _1$ are logic expressions;
- if $\varphi$ is a logic expression, and *v* is a variable, then $\exists v:\varphi$ and $\forall v:\varphi$ are logic expressions.

**Fig. 6:**
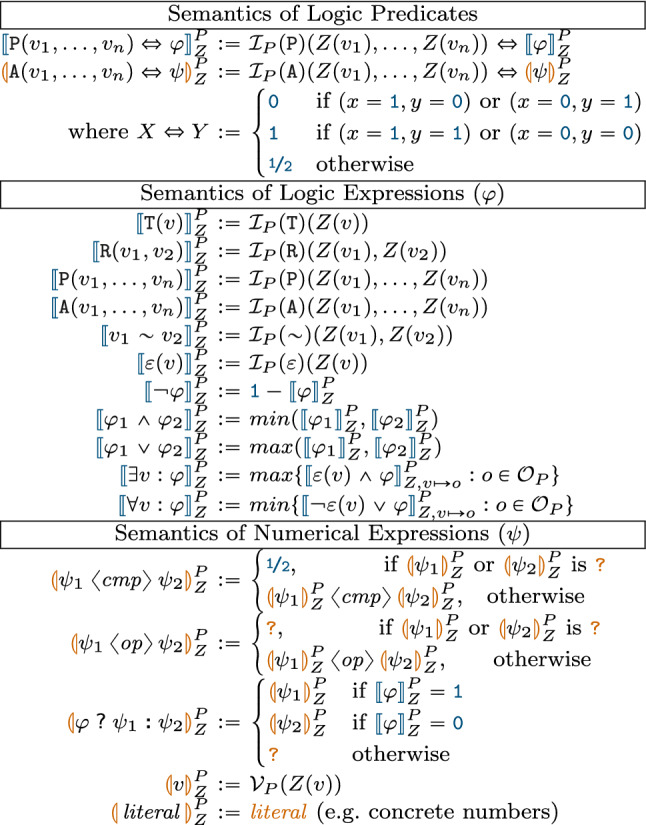
Inductive semantics of graph predicates

For each attribute constraint, we derive *attribute predicates* (as helpers) by reification to enable seamless interaction between structural and attribute solvers along a compatibility (if and only if) operator $\Leftrightarrow$ (see Fig. 6). In case of numbers, such an attribute predicate is tied to a *numerical predicate* defined as  where $\psi$ is constructed from *numerical expressions*. The expressiveness of those expressions is limited by the background theories of the underlying backend numeric solver. Here we define a core language of basic arithmetical expressions, which is supported by a wide range of numeric solvers:

- each variable *v*, constant symbol and literal (concrete number) *c* is a numerical expression,
- if $\psi _1$ and $\psi _2$ are numerical expressions, then $\psi _1 + \psi _2$, $\psi _1 - \psi _2$, $\psi _1 \times \psi _2$ and $\psi _1 \div \psi _2$ are numerical expressions.
- if $\psi _1$ and $\psi _2$ are numerical expressions, then $\psi _1 < \psi _2$, $\psi _1 > \psi _2$, $\psi _1 \ge \psi _2$, $\psi _1 \le \psi _2$, $\psi _1 = \psi _2$, $\psi _1 \ne \psi _2$ are numerical predicates.
- if $\varphi$ is a logic expression, and $\psi _1$ and $\psi _2$ are numerical expressions, then  is a numerical expression (standing for “if $\varphi$ then $\psi _1$ else $\psi _2$”, commonly used in programming languages).

#### Example 3

Pattern parentTooYoung(child, parent) of Fig. 5 is formalized as the following logic predicate:
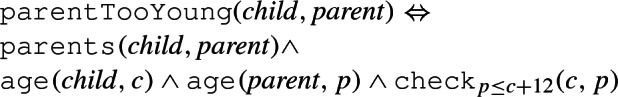


 is a numerical predicate.

Later such predicates will help communicate between different solvers, e.g., if  is found to be  by the graph solver for some members $c_1$ and $p_1$, then the numerical predicate $p_1\le c_1+12$ needs to be enforced by a numeric solver for the respective data objects and vice versa.

*Semantics*

A logic predicate  can be evaluated on a partial model *P* along a variable binding  (denoted as ), which can result in three truth values: ,  or . The inductive semantic rules of evaluating a logic expression are listed in Fig. 6. Note that $\mathop {min}$ and $\mathop {max}$ take the numeric minimum and maximum values of ,  and .

A numerical predicate  can be evaluated on a partial model *P* along variable binding  (denoted as ) with a result of ,  or . The inductive semantic rules of logic expressions are listed in Fig. 6. Note that  means the truth value of numerical comparison  (e.g., $3<5$ is ), while  means the numeric value of the result of an operation $\langle {\textit{op}}\rangle$ (e.g., $3+5$ is ).

*Constraint approximation* When a predicate is evaluated on a partial model, the 3-valued semantics of constraint evaluation guarantees that certain (over- and under-approximation) properties hold for all potential refinements or concretizations of the partial model. For all logic and numerical predicates $\varphi$ and $\psi$, , thus:

- **Logic under-approximation**: If  in a partial model *P*, then  in any partial model *Q* where .
- **Numeric under-approximation**: If  in a partial model *P*, then  in any partial model *Q* where .
- **Logic over-approximation**: If  in a partial model *Q*, then  in a partial model *P* where .
- **Numeric under-approximation**: If  in a partial model *Q*, then  in a partial model *P* where .

These properties ensure that model generation is a monotonous derivation sequence of partial models which starts from the most abstract partial model where all predicate constraints are evaluated to . As the partial model is refined, more and more predicate values are evaluated to either  or . The under-approximation lemmas ensure that when an error predicate is evaluated to , it will remain ; thus, exploration branch can be terminated without loss of completeness [87]. The over-approximation lemmas assure that if a partial model can be refined to a concrete model where error predicate is , then it will not be dropped.

## Model generation with refinement

### Functional overview

Our framework takes the following inputs:

1. the signature of a domain $\langle \Sigma ,\alpha \rangle$ (derived from a metamodel or ontology) with structural logic symbols  and numerical attribute symbols ,
2. a logic theory consisting of the negation of the error predicates and the compatibility of the predicate symbols with their definition (i.e., the axioms):
3. some search parameters (e.g., the required size, or the required number of models).

The output of the generator is a sequence of models $M_1, \ldots , M_m$, where each $M_i$ is *consistent*, which means

1. a regular concrete model of $\langle \Sigma ,\alpha \rangle$;
2. consistent with ${\mathcal {T}}$ (), i.e., for any *i*, *j*, no error predicates have a match  , and all predicates  and  are compatible with their definition  and ;
3. adheres to search parameters (e.g., ).

**Fig. 7:**
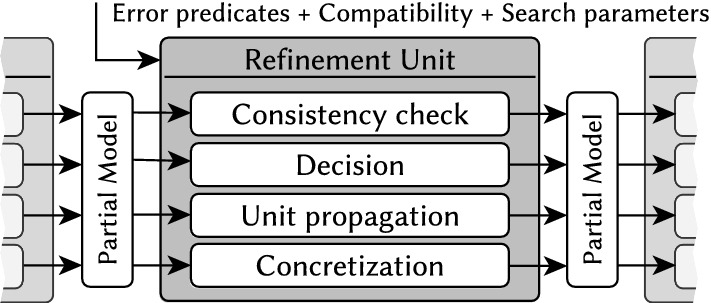
Schematic overview of a refinement unit

The model generator combines individual *refinement units* to solve structural and numerical problems. Each refinement unit analyzes a partial model (which is an intermediate state of the model generation), and it collaborates with other units by refining it. This is in conceptual analogy with the interaction of background theories in SMT-solvers [50, 51]. A refinement unit provides four main functionalities (see Fig. 7):

**Fig. 8:**
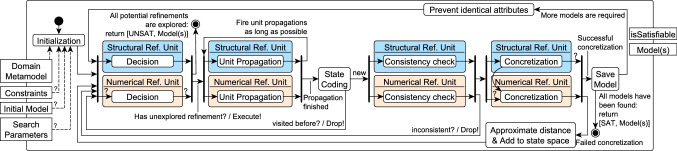
Overview of the default state space exploration strategy for model generation

- **Consistency check:** The refinement unit evaluates whether a partial model may satisfy the target theory (thus it can be potentially completed to a consistent model), or it surely violates it (thus no refinement is ever consistent).
- **Decision**: The unit makes an atomic decision by a single refinement in the partial model (e.g., adding an edge by setting a  value to ) which is consistent with the target theory. This new information makes the model more concrete, thus reducing the number of potential solutions.
- **Unit propagation:** After a decision, the unit executes further refinements necessitated by the consequences of previous refinements wrt. the target theory without introducing new information or excluding potential solutions. This step automatically does necessary refinements on the partial model without making any decisions.
- **Concretization:** Finally, the unit attempts to complete the partial model by setting all uncertain  edges to , and checks if the concrete model is consistent with the target theory or not.

In this paper, we combine two of such refinements units: We reuse a graph solver [71] as  to efficiently generate the structural part of models to reason about . Moreover, we propose a novel  that uses two efficient backend SMT-solvers (Z3 [17] and dReal [23]) to solve the numerical problems reasoning about . The refinement units interact with each other bidirectionally via the refinement of partial models: The  refines truth values on attribute predicates (based on the structural part of the error predicates), which need to be respected by the . Symmetrically, the  can refine attribute predicates (based on the numerical part of the error predicates), which need to be respected by the  in turn.

In case of circular dependencies between structural and numeric constraints, the  first enumerates all possible non-isomorphic structures, then the  attempts to resolve the attribute values, given the graph structure. Potential conflicts between refinement units are handled during consistency checks in subsequent exploration steps, as described in Sect. 4.2. Nevertheless, more complex decision procedures may be implemented to handle such circular constraints.

### Default exploration strategy by refinements

Our model generation framework derives models by exploring the search space of partial models along refinements carried out by refinement units. Thus, the size of the partial models is continuously growing up to a designated size, while the default exploration strategy aims to intelligently minimize the search space. The detailed steps of this default strategy are shown in Fig. 8.

Our framework takes as input a domain metamodel provided by an engineer. Optionally, engineers may also provide as input *additional logic constraints* and an *initial partial model*, as well as some *search parameters*.

**0. Initialization:** First, we initialize our search space with an initial partial model. This is derived either from an existing initial model provided as input (thus each solution will contain this seed model as a submodel), or it can be the *most general partial model*  where  has a single element,  is  for every symbol, and .

**1. Decision:** Next, we select an unexplored decision candidate proposed by a refinement unit, and execute it to refine the partial model by adding new nodes and edges, or by populating a data object with a concrete value. In the default strategy, this decision step is executed mainly by the structural refinement unit which has more impact on model generation. If no decision candidates are left unexplored, the search concludes with an UNSAT result and returns the models that have been saved during previous iterations, if any.

**2. Unit propagation:** After a decision, the framework executes unit propagation in all refinement units until a fixpoint is reached in order to propagate all consequences of the decision.

**3. State coding:** The search can reach isomorphic partial models along multiple trajectories. To prevent the repeated exploration of the same state, a state code is calculated and stored for a new partial model by using shape-based graph isomorphism checking [55, 56]. If exploration detects that a partial model has already been explored, it drops the partial model and continues search from another state. Otherwise, the framework calculates the state code of the newly explored partial model and continues with its evaluation.

**4. Consistency check:** Next, each refinement unit checks whether the partial model contains any inconsistencies that cannot be repaired.  evaluates the (logic) under-approximation of the error predicates (see Sect. 3.4), which can detect irreparable structural errors. The  carries out a satisfiability check of the numeric constraint determined by a call to the numeric solver.

**5. Concretization:** Then, the framework tries to concretize the partial model to a fully defined solution candidate by resolving all uncertainties, and checks its compliance with the target theory and model size. If no violations are found and the model reaches the target size, then the instance model is saved as a solution. (Thus, consistency is ensured for all solutions.) If this concretization fails, it indicates that something is missing from the model, so the refinement process continues.

**6.1. Approximate distance & Add to state space:** When a partial model is refined, our framework estimates its distance from a solution [44]. This heuristic is based on the number of missing objects and the number of violations in its concretization. Then, the new partial model is added to the search space of unexplored decisions where the exploration continues at **1. Decision**.

*Further heuristics:* For selecting the next unexplored decision to refine, we use a combined exploration strategy with best-first search heuristic, backtracking, backjumping and random restarts with an advanced design space exploration framework [31, 71].

**6.2. Save Model: ** If concretization is successful, the instance model is saved as a solution. At this stage, if the required number of models has been reached, the search concludes with a SAT result and returns all the models that have been saved. Otherwise, the refinement process continues.

**7. Prevent identical attributes:** After finding a concrete instance model, we avoid finding duplicates during future iterations by adding constraints to the logic theory. These constraints ensure that the numeric attribute assignments are not identical to assignments provided for a previous model. Exploration then continues at **1. Decision**.

*Diversity in attributes:* The feedback loop provided by the **7. Prevent identical attributes** step may also be used to increase diversity in attribute values. For example, instead of just preventing duplicate numeric solutions, one may enforce certain domain-specific coverage criteria during numeric concretization (e.g., solutions must be generated such that they are evenly distributed over an interval). One may also implement equivalence classes for attribute values through qualitative abstractions to further improve both structural and numeric diversity. We plan to address these enhancements as part of our future work.

**Fig. 9:**
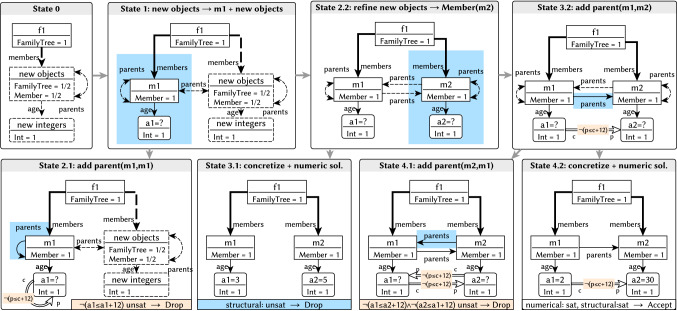
Sample realization of the *default strategy* for state space exploration

#### Example 4

Figure 9 illustrates a model generation run to derive a family tree. Search is initialized with a $f1$ as root and two (abstract) objects to represent new objects and new integers.

**State 1** highlights the execution of a decision that splits the new object and the new integer, creating a new *m*1 with its undefined  attribute.

In **State 2.1**, a loop  edge is added as a decision. When investigating error predicate , the search reveals that all conditions of the error predicate are surely satisfied on objects *m*1 and *a*1 except for attribute predicate . Therefore, the  can refine the partial model by setting  to  without excluding any valid refinements, which implies that . The  (with the help of an underlying numeric solver) can detect that no value ${\mathcal {V}}_{S2.1}(a1)$ can be bound to object *a*1 such that ${\mathcal {V}}_{S2.1}(a1)\le {\mathcal {V}}_{S2.1}(a1)+12$ is false; therefore, the model cannot be finished to a consistent model; thus, it can be safely dropped.

In **State 2.2**, a new *m*2 is added to the , and the framework attempts to concretize the model by resolving all uncertainty in **State 3.1**. First, the  concretizes in the structural part of the model, all  values are set to  (e.g., all the potential  edges disappear). Then, sample valid values are generated for the attributes by the . When the concretization is checked, error pattern $(m1,m2)$ indicates that there are missing  edges, so the framework drops the concretization but continues to explore **State 2.2**.

Eventually, after adding a  edge in **State 3.2**, the framework is able to concretize a (consistent) model in **State 4.2** that satisfies the target theory. In this case, we only require a single output instance model; thus, the search terminates.

### Custom exploration strategies

The default exploration strategy in Sect. 4.2 handles model generation domain-independently. As such, it cannot exploit the specificities of a modeling domain to accelerate state space exploration. Furthermore, the default approach handles the constantly growing partial model representation as a whole during refinements. Although this does simplify the process, it also poses a scalability challenge for complex model generation tasks like in the Crossing Scenario domain.

To address these issues, typical theorem proving practices expose the internal decision processes and enable users to define custom search space exploration strategies. The approach proposed in [24] explicitly provides preferred states as hints to guide exploration towards preferred search space regions. The authors of [59] place domain-independent conditions to restrict the instantiation of quantifiers during search. The Z3 SMT solver [17] provides tactics and probes, as well as combinators to allow significant customization of underlying decision processes. In all cases, the proposed customizations allow users to strategically restrict the search space, thus guiding exploration.

Here, we adapt similar concepts to model generation and propose an approach to define domain-specific, custom exploration strategies. These strategies are used to restrict the search space and guide exploration towards desired search space regions. Users may specify strategies (based on domain knowledge and requirements), which can split the modeling domain into fragments^2^ that are handled consecutively in accordance with the divide-and-conquer principle. An example of such a strategy is proposed in [53] and is applied to the context of test generation for Software Product Lines.

**Syntax:** A *custom strategy* is composed of phases, where each phase is responsible for the creation of a concrete fragment of the partial model. A phase may contain one  (pertaining to structural decisions), followed potentially by a  (pertaining to numeric decisions). Additionally, a strategy may include a final subphase that is not associated with any phase and that marks the end of the strategy. Furthermore, each phase contains a set of *relaxed constraints* (not checked) which are excluded, while the exploration is at the corresponding phase. Finally, phases may also contain a set of *preferred numeric solver*, only one of which may be used at each phase.

#### Definition 5

(Custom strategy) For a set *D* of decisions, a set *C* of constraints and a set *N* of numeric solvers, a *custom strategy* is defined by a deterministic control flow graph $CFG = \langle S,s, T\rangle$, where

- *S* represents a finite set of *subphases*,
- $s\in S$ is the *initial subphase*,
- $T\subseteq S \times 2^{D} \times 2^{C} \times 2^{N} \times S$ represents the set of *transitions* where a tuple $( src , dec , rel , num , trg )\in T$ is composed of the source subphase $src$, the set of allowed decisions $dec$, the set of relaxed constraints $rel$, the set of preferred numeric solvers $num$, and the target subphase $trg$.

Additionally, given a subphase $c \in S$ and the set of all its outgoing transitions $\{x_0, \ldots , x_m\} \subset T$, where $x_i = (c,d_i,r_i,n_i,t_i)$, deterministic execution is enforced by $\{ d_j \cap d_k = \emptyset : 0 \le j \le k \le m\}$.

**Fig. 10:**
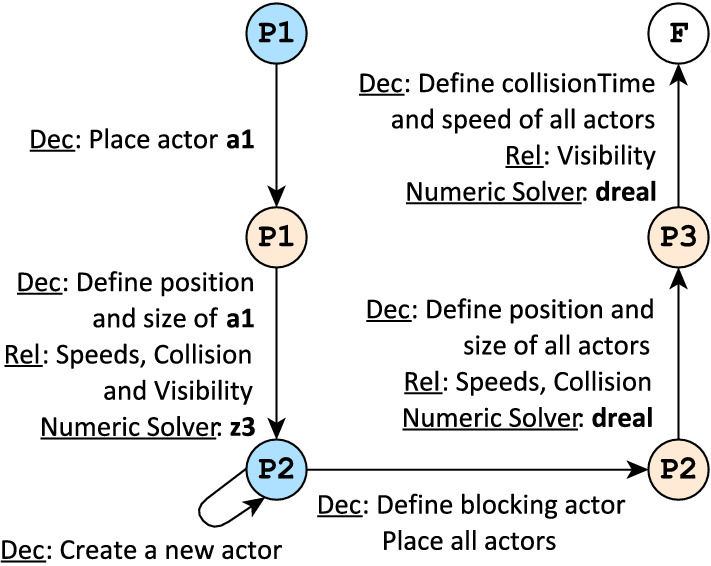
Control flow graph defining a 3-phase *custom strategy* for Crossing Scenario

#### Example 5

Figure 10 defines a 3-phase custom strategy for the domain of critical traffic scenarios. Subphases are named according to their corresponding phase, and colored according to the nature of the decisions in their outgoing transitions (  or ). The control flow graph also contains a final subphase F. Transitions are labeled with corresponding *decisions* (Dec), *relaxed constraints* (Rel) and *preferred numeric solver* (Numeric Solver), where applicable.

Figure 10 illustrates a sample strategy designed *specifically* for the domain of traffic scenarios. A different strategy could be defined for the family tree domain (see Sect. 3.1), which refer to concepts and attributes of that domain (e.g., family members or parenthood instead of vehicle speed and visibility). As such, the main structural constituents of an exploration strategy (i.e., decisions, relaxed constraints and preferred number solver) will always refer to domain-specific concepts.

**Semantics:** A model generation run using a custom strategy corresponds to a traversal of the associated control flow graph which is defined by a sequence of transitions traversals. Semantically, a transition traversal corresponds to an iteration of the default exploration strategy. Thus, a traversal potentially involves all four refinement unit functionalities of Fig. 8.

A custom strategy can restrict the default strategy, but cannot extend it: *relaxed constraints* are excluded, allowed *decisions* are restricted and a *preferred numeric solver* is used for numeric decisions. As a result, each transition traversal addresses only a specific fragment of the modeling domain. Therefore, a sequence of transition traversals may address the entire modeling domain through a divide-and-conquer approach.

#### Definition 6

(Execution of custom strategies) Let  be a custom strategy over a set of decisions , constraints , and numeric solvers . Iteration *i* from state $src$ to state $trg$ conforms with $CFG$, if there is a transition , where:

- the refinement applies a decision $d\in dec$,
- the refinement excludes constraints in $rel$ during consistency check
- the refinement uses a numeric solver $n\in num$

An iteration sequence $i_1,\ldots ,i_n$ conforms with $CFG$, if:

- iteration $i_1$ from initial state *s* conforms with $CFG$,
- for each pair $(i_j, i_{j+1}) : 1 \le j \le {n-1}$ iteration $i_j$ to state *x* conforms with $CFG$, and $i_{j+1}$ from state *x* conforms with $CFG$.

**Fig. 11:**
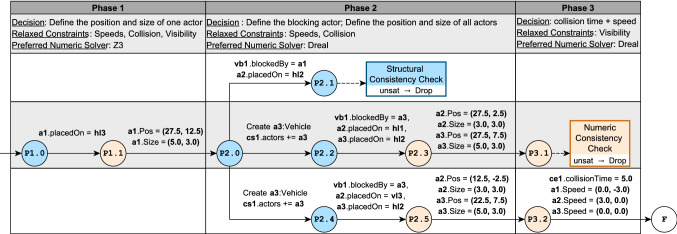
Sample traversal of the control flow graph defined shown in Fig. 10

**Fig. 12:**
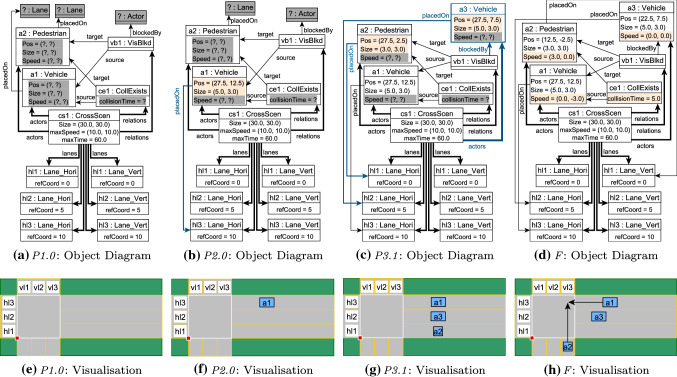
Object diagrams and visualizations for selected nodes in the CFG traversal shown in Fig. 11

#### Example 6

Figure 11 illustrates a traversal of the CFG for the 3-phase custom strategy of Crossing Scenario in Fig. 10. Each column corresponds to a phase and includes the associated subphases and transitions.

Key subphases are accompanied by corresponding object diagrams (concretized from the underlying partial model) and visualizations in Fig. 12. The objects diagrams show existing (filled) attributes and structural components in black. Unknown partial model elements, such as attribute values and relations, are highlighted in dark gray. Elements that have been defined as a result of decisions executed during the previous phase are colored according to their type (  or ). These elements are also explicitly indicated in the corresponding phase blocks of Fig. 11.

**P1.0** is the initial subphase, where the underlying partial model (in Fig. 12e) contains an empty road map with 3 vertical lanes and 3 horizontal lanes. Figure 12a shows two unplaced actors a1 and a2 that have their vision blocked and that must eventually collide. At this stage, actor a1 is placed on horizontal lane hl3, then assigned concrete numeric position and size values.

At **P2.0**, the strategy moves to **P2.1** by setting a1 as the vision blocking actor between itself and a2. After a structural consistency check, this partial model is dropped, and the exploration backtracks.

**P2.0** is reached for a *second time*. At this stage, a new actor a3 is created and the exploration moves to **P2.2**. It sets a3 as the vision-blocking actor and places actors a2 and a3 on horizontal lanes. Once the structural consistency checks succeed, the exploration moves to **P2.3** and assigns concrete numeric position and size values to a2 and a3. The exploration reaches **P3.1**, where a3 is clearly blocking the vision between a1 and a2, as seen in Fig. 12g. However, we notice that since all actors can only move in a horizontal direction, there is no chance for any of them to collide. As a result, when the exploration tries to enforce the collision between actors a1 and a2 in **P3.1**, the consistency check fails and the exploration backtracks.

**P2.0** is reached for a *third time*. As in the previous traversal, a new actor a3 is created, set as the blocking actor and placed on a horizontal lane. However, actor a2 is placed on a vertical lane. Once concrete position and size values are provided to a2 and a3, the exploration moves to **P3.2**. At this stage, a possible collision does exist between actors a1 and a2, as seen in Fig. 12h. The corresponding numeric decisions are made and the exploration moves to state **F**, which outputs the concrete model shown in Fig. 12d.

### Summary

Our framework constructs models by applying partial model refinements proposed by refinement units. This paper uses a combination of three refinement units:

- a graph solver [71] as ;
- the Z3 SMT solver [17] as ;
- the dReal solver [23] as .

The framework combines the refinement units to explore the search space of potential refinements using different strategies.

- *Default strategy* follows a general purpose execution plan detailed in Sect. 4.2.
- A *custom strategy* restricts the exploration with domain-specific hints to improve performance as illustrated on generating traffic layouts in Sect. 4.3.
- Finally, a *combined strategy* applies a custom strategy first, and if it fails, the exploration backs of to the default exploration strategy. Therefore, the custom strategy is used only as a *heuristic* to select the preferred refinements, if possible.

## Structural and numerical refinement units

In this section, we summarize the  and  used in this paper. We also detail a mapping from a partial model to a numerical problem handled by an underlying numeric solver.

### Structural refinements by a graph solver

The structural *consistency* of a partial model can be verified by checking the compatibility of all predicates  as . If a predicate is incompatible with its definition, or an error predicate is satisfied, the partial model is inconsistent (see Sect. 3.4).

Our framework operates on a graph representation of partial models (without a mapping to a logic solver); thus, structural predicates are evaluated directly on this graph representation. The query rewriting technique [72] enables to efficiently evaluate the 3-valued semantics of logic predicates  by a high-performance incremental model query engine [85, 86], which caches and maintains the truth values of logic predicates during exploration.

Structural refinements are implemented by graph transformations [71, 87]. *Decisions* are simple transformation rules that rewrite a single  value to a  in the partial model, or an equivalence predicate  to  to split an object to two (like $m1$ is separated from *new object* in **State 1**). On the other hand, *concretization* rewrites all  values to , and self-equivalences to .

The compatibility of predicate symbols is checked by structural *unit propagation rules*, which are derived from error predicates to refine a partial model when needed to avoid a match of an error predicate. We rely on two kinds of unit propagation rules:

- We derive unit propagation rules from the structural constraints imposed by the metamodel to enforce type hierarchy, multiplicities, inverse references, and containment hierarchy [71]. For example, when a new  is created, a new  is also created with an  predicate between them.
- Unit propagation rules are derived from each error predicate  to check if a  (or ) value would satisfy the error predicate . In such cases, the value is refined to the opposite  (or ). Such unit propagation rules may add numerical implications of error predicates.

### Numerical refinements by numeric solvers

The  is responsible for maintaining the compatibility of numeric constraints and attribute predicates, checking consistency of numeric constraints, and deriving concrete numeric values.

Numerical refinement is based on a purely numerical interface of a partial model. Let *P* be a partial model with attribute predicates . Let  denote the set of data objects where . The *numerical interface*  consists of the objects in , and of the numerical predicate values of the logic interpretations .

**Fig. 13:**
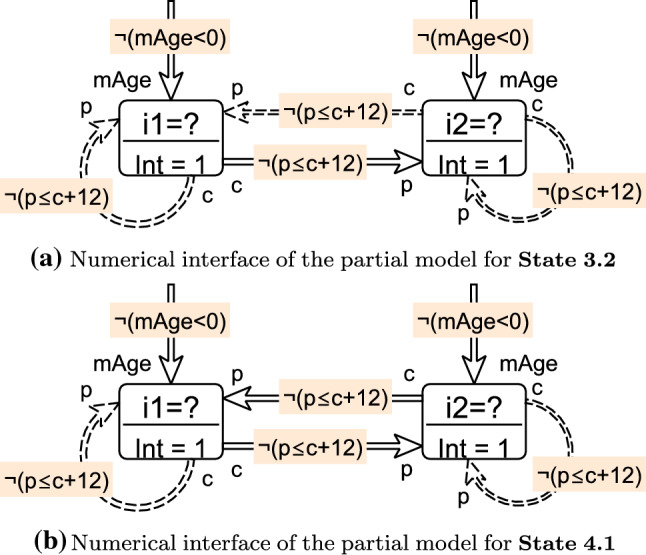
Numerical interfaces for two states in Fig. 9

#### Example 7

We extract numerical interfaces of the partial models for **State 3.2** and for **State 4.1** in Fig. 9. These numerical interfaces are shown in Fig. 13, which contains edges, representing attribute predicates, labeled with the negations of the corresponding numeric constraint. Constraint negations are more common within partial models as their *violating cases* are included in the modeling domain. For both interfaces, the numeric objects are . The numeric values for interpretations  and  of predicates  and  are:
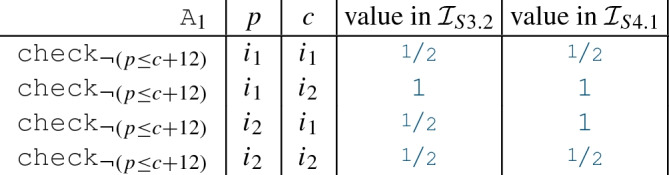

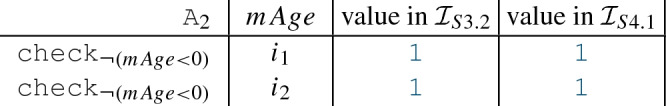


A numeric solver is called on the numerical interface  of a partial model *P*. The solver call returns a truth value for the satisfiability of . For satisfiable numerical interfaces, the solver also returns a numeric value assignment for each object in .

A *consistency check* for partial models involves a numeric solver call where only the satisfiability of the numerical interface is verified without any value assignments. An unsatisfiable numerical interface  implies that the partial model *P* cannot be completed with consistent numeric values; thus, it can be dropped.

If the interface is satisfiable, numeric value assignments for elements in  are used to complete partial models by providing an interpretation for all unbounded data objects during *concretization*. Moreover, fixing potential values for certain data objects can be used as a *decision*. Numerical interfaces formulated on real numbers need special attention. A numeric solver may provide either *exact* or *approximated* solutions.

- An *exact solution* is a mathematically precise solution to a given numerical interface.
- An *approximated solution* is a numeric solution that satisfies a $\delta$-perturbed form of the input formula (as defined in [22]), where the preciseness is controlled with a $\delta$ approximation parameter.

The numerical consequences of the constructed  can be used to refine a partial model during *unit propagation*. In our framework, three kinds of unit propagation operations are supported:

- *Values*$\rightarrow$*Predicate.* When the values of numeric objects $o_1,...,o_n$ are known in a partial model (i.e., ), then the truth value of a predicate  can be evaluated and updated in the model. This step can be done without calling the numeric solver, as the numerical expression can be evaluated pragmatically (e.g.,  for  and  is ).
- *Predicate*$\rightarrow$*Predicate.* If an attribute predicate  has an unknown value , and  is numerically inconsistent, then  can be refined to . Similarly, if  is inconsistent, the attribute can be refined to . For example, in partial model *S*3.2, a value  for predicate  on $i_1, i_1$ would cause numerical inconsistency, so the framework can refine it to . (In our case studies, this step was impractical thus this feature was not used.)
- *Predicate*$\rightarrow$*Values.* If there is only a single solution *x* for a numeric object *o*, then the unique value of can be set in an unit propagation step ${\mathcal {V}}_P(o)=x$. For example, if , then  would be the only solution, and it can be refined in the partial model as a unit propagation.

### Mapping of numerical interfaces

In this section, we describe how a numerical interface  of a partial model *P* is mapped to a *numerical problem* that is handled by an underlying numeric solver. As discussed in Sect. 5.2,  is defined over the set of data objects . It also contains the numerical predicate values of the logic interpretations  where  are attribute predicates contained in *P* and defined by .

Each data object  is mapped to a numeric variable  denoting its potential value. If , then the type of this variable is integer, while if , then it is real. The numerical problem derived from  is defined over those variables.

A numerical problem is constructed as the conjunction of numerical assertions as follows. If the value of *o* is already known in the partial model (), then we assert its value as a numerical equation: . Additionally, for each attribute constraint  in *P*, we assert its definition  for all data objects:

- If , then
- If , then
- If , then nothing is asserted.

This mapping extends our previous work [69], where we provide a complete mapping from structural constraints to FOL.

#### Example 8

We illustrate this mapping for the numerical interface shown in Fig. 13b, which corresponds to the partial model for **State 4.1** of Fig. 9. The values for the interpretation  of relevant attribute predicates are indicated in Sect. 7.

**Fig. 14:**
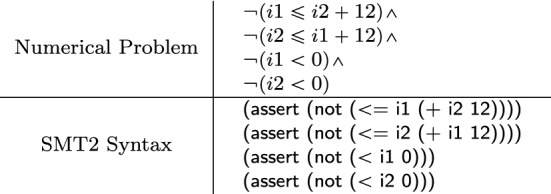
Mapping outputs for the numerical interface shown in Fig. 13b

Mapping outputs are shown in Fig. 14. We include the logic formulation of the numerical interface as a *Numerical Problem*. Furthermore, we provide a translation of the numerical problem into the concrete *SMT2* syntax that is handled by numeric solvers such as Z3. We may notice that we only include (negated) assertions for attribute constraints  with logic interpretation , while predicates with interpretation  are disregarded.

### Soundness and completeness

With the combination of the  and , our proposed approach generates models with numerical attributes using partial model refinement. For an input domain ${\langle \Sigma ,\alpha \rangle }$, theory (constraints) ${\mathcal {T}}$ and the required number and size of models, it generates a sequence of models $M_1,\ldots ,M_n$.

In this section, we evaluate the theoretical properties and guarantees of our approach in different configurations. The model generator can be executed using one of the three following strategies:

- *Default strategy* (detailed in Sect. 4.2)
- *Custom strategy* (detailed in Sect. 4.3)
- *Combined strategy* (detailed in Sect. 4.4)

For , the model generator has two options:

- use *exact numeric solvers* only (like Z3).
- use *approximate numeric solvers* (like dReal).

With respect to decidability of the numerical problem:

- If the numerical fragment is *decidable*, then the  always terminates.
- If the numerical fragment is *undecidable*, then the  may not terminate for certain numerical problems.

#### Definition 7

(Consistency) A model generation approach is *(approximately) consistent*, if every model in the generated sequence $M_i\in \{M_1,\ldots ,M_n\}$ is consistent (approximately) satisfies the theory ${\mathcal {T}}$ () and adheres to the search parameters.

According to this definition, our model generation is *consistent* if it uses exact numeric solvers, and it is *approximately consistent*, if it uses approximate numeric solvers. This is guaranteed by the direct evaluation of the error predicates and compatibility predicates on the final stage of model refinement with the underlying refinement units (see **5. Concretization** in Sect. 4.2). Consistency is not influenced by the decidability of the numerical problem, or the strategy.

To discuss completeness, model equivalence is defined first.

#### Definition 8

(Model isomorphism) Two partial models *P* and *Q* are *structurally isomorphic*, if there is a bijective function , where for each n-ary symbol $s\in \Sigma$ and for all objects :Partial models *P* and *Q* are *isomorphic*, if for each object : ${\mathcal {V}}_{P}(o) = {\mathcal {V}}_{P}(m(o))$ is also satisfied. Two models *P* and *Q* are *(structurally) different*, if they are not (structurally) isomorphic.

Therefore, we can define completeness properties for the model generator.

#### Definition 9

(Structural completeness) A model generation approach is *structurally complete*, if for the given theory ${\mathcal {T}}$ and a search parameters, it can generate a sequence $M_I\in \{M_1,\ldots ,M_n\}$ that contains all structurally different and consistent models.

Our *default exploration strategy* approach is *structurally complete* on *decidable* numerical refinement unit: For a given scope (size), it is able to generate all models with different graph structures, which is ensured by the approximation lemmas in Sect. 3.4 and in [71, 87]. We intentionally avoid fulfilling *numerical completeness*, since even simple models could have potentially infinite number of attribute bindings. A *custom strategy restricts* the search space to improve the performance of model generation at the cost of completeness guarantees. As the *combined strategy* eventually terminates, and continues with the *default exploration strategy*, it has the same completeness guarantee as *default exploration strategy*. If the numerical fragment is not decidable, then we cannot ensure that the numeric solver is able to provide numeric solutions for each structure, and we cannot guarantee completeness.

## Evaluation

We conducted various measurements to address the following research questions:

**RQ1**: How do the different exploration steps contribute to the execution time for generating models?
**RQ2**: How does model generation scale to derive large models with structural and attribute constraints?
**RQ3**: How do various exploration strategies influence the efficiency of model generation?
**RQ4**: How structurally diverse are synthetic models?

### Target domains

We perform model generation campaigns in four complex case studies. The target domain artifacts, output models and measurement results are available on GitHub^3^.

: The *FamilyTree* domain is presented in Sect. 3.1 as our running example. We use the metamodel shown in Fig. 4 which captures parenthood relations and the age of family tree members (with 2 classes, 3 references and 1 numerical attribute). Furthermore, 3 constraints are defined as graph predicates that place structural and numerical restrictions on family tree members. The initial model used for model generation contains a single  node. While this domain looks simple, there is a subtle mutual dependency between structural and attribute constraints, which provides extra challenges for the interaction of different solvers.

: The *Satellite* domain (introduced in [32]) represents *interferometry mission architectures* used for space mission planning at NASA. Such an architecture consists of collaborating satellites and radio communication between them, which are captured by a metamodel with 15 classes, 5 references and 2 numerical attributes. Additionally, 18 constraints are defined as graph predicates to capture restrictions on collaborating satellites. The initial model contains a single root node as the starting point for model generation.

: The *Taxation* domain (used in [81, 82]) represents the personal income tax management application used by the Government of Luxembourg. We reused the original metamodel which contains 54 classes (including 15 Enum classes), 52 relations and 92 attributes, 44 of which are numerical. Additionally, we replicated the OCL constraints used in [82] as graph predicates.

To independently replicate the case study of [82] in a pure EMF context with strict containment hierarchy (instead of UML), we include a  class in the metamodel that contains instances of the  class, which was the root class of the original *Taxation* metamodel. This allows the instantiation of multiple  instances within the same model generation task. To enforce the same number of objects, we include an initial model containing a predefined number of  instances and we prevent the generation of further instances of that class as in [82].

: The *CrossingScenario* domain is presented in Sect. 2 as our motivating example. We identify two variants of this domain for our experimental evaluation:  is used for **RQ1** and  used for **RQ3**. Both variants use the metamodel in Fig. 2 to capture the actors and lanes of a traffic scenario, as well as certain spatial and temporal relations between actors (with 10 classes, 7 references and 13 numerical attributes).

 includes 10 constraints defined as graph predicates that place structural and numerical restrictions on the positioning and size of actors with respect to their corresponding lanes. Additionally, we include a (quadratic) constraint to set a minimum Euclidean distance between any pair of actors in the model. The initial model for this variant includes 5 vertical lanes and 5 horizontal lanes. The model generation challenge is to populate the existing lanes by placing new  objects (but without extra lanes or relations).

 includes 32 constraints defined as graph predicates that place restrictions on all components of the metamodel. We incorporate complex numeric constraints such as quadratic inequalities, non-constant divisions and numeric *if-then-else* blocks. The initial model for this variant includes 4 vertical lanes and 4 horizontal lanes, as well as two black actors (with empty attributes) which are connected by a  relation and a  relation. The model generation task consists of placing the actors on specific lanes and filling their attribute values such that the constraints are satisfied. Additional actors may be generated if required. This variation provides a significant model generation challenge not only due to the complexity of the included numeric constraints, but also due to their mutual dependencies with structural constraints, which necessitates a bidirectional interaction between the underlying solvers.

**General setup:** To account for warm-up effects and memory handling of the Java 8 VM, an initial model generation task is performed before the actual measurements and the garbage collector is called explicitly between runs. We performed the measurements on an enterprise server^4^.

### RQ1: cost of exploration phases

**Measurement setup:** We perform measurements in all four domains to compare runtimes and their distribution between the different phases of model generation. For each domain, we run measurements twice, with different underlying numeric solvers (Z3 and dReal). Note that the numeric solvers are only used to assess the numeric constraints of the model generation task and not the structural constraint as poor scalability was reported in [5, 69] for the latter.

We generate models with an increasing minimum model size of 20, 40, 60, 80 and 100. The range for numeric values was not bounded a priori. We exclude larger model sizes to ensure high success rates and to enable cross-domain comparison of execution phases. For the  domain, the initial model contains one instance of the  class for every 20 generated nodes (which is the typical size of a household in the models generated in [82]) to balance the difficulty of model generation regardless of the target model size.

Initial measurements showed that the complexity of numeric constraints in  causes low success rates even for small models. Thus, we generate models containing only up to 21 nodes (with a step size of 3).

We execute 10 runs per target model size and take the median runtime values. For each model generator run, we aim to produce the first 10 models within a timeout of 5 minutes. For the  domain, we excluded the generation of additional models due to the low success rates.

**Fig. 15:**
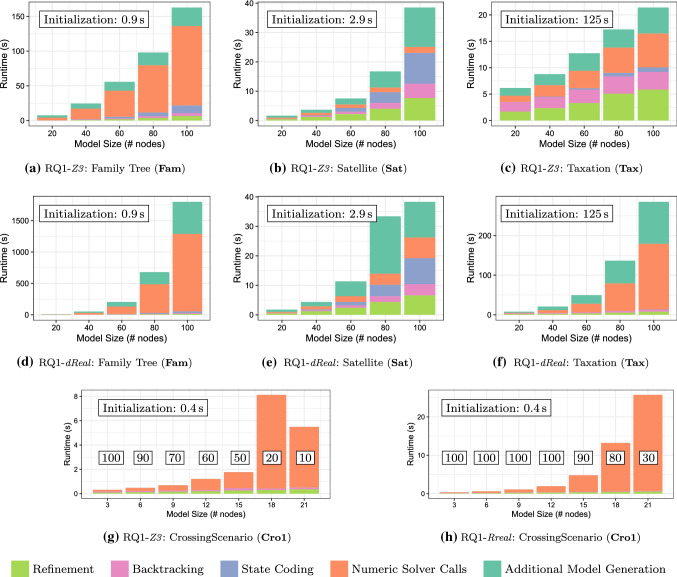
Runtimes of different exploration steps when generating models of increasing size

**Analysis of results:** The decomposition of runtime measurements for all four domains is shown in Fig. 15. Each phase of model generation is represented by a different color. The initialization phase (0.9 seconds for , 3.5 seconds for , 150 seconds for , 0.4 seconds for ) is a one-time penalty which is proportional to the size of the metamodel and to the number of additional WF constraints.

In the  domain, the runtime is dominated by numeric solver calls. This is attributed to the fact that this domain needs to enforce a global structural constraint (families must have an acyclic graph structure with respect to the parenthood relation) by solving numeric constraints, while the numeric constraints of other domains are dominantly local (e.g., to fill attribute values). However, extra cost of generating subsequent models is low.

In the  case study, generating the first model takes less than 30 seconds (dominated by the time required for state encoding), but the cost of incrementally generating the next model is relatively larger. For the target model sizes, execution times in the  case study are still mostly dominated by the initialization phase due to the large metamodel and numerous constraints of the domain. However, we do notice that a significant portion of the execution time is dedicated to numeric solver calls, due to the large quantity of numeric constraints.

In all of the above cases, we notice that dReal requires more time than Z3 (by an order of magnitude for  and ) to handle the numeric constraints. Thus, we conclude that for simple numeric constraints, Z3 is the more performant underlying numeric solver.

However, we notice that dReal is the better performing numeric solver for the  domain. Figure 15g and 15h shows that the runtime is significantly dominated by numeric solver calls, as expected. The figures also show the decreasing success rate for model generation runs for both numeric solvers. We notice that although runtimes for Z3 are slightly faster than for dReal, we can see that success rates of Z3 decrease more rapidly. This is attributed to the underlying background theories used in dReal, which make it more suitable for complex, nonlinear constraints such as those in .
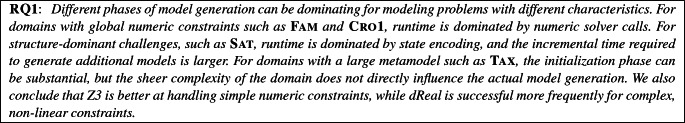


### RQ2: scalability of model generation

**Fig. 16:**
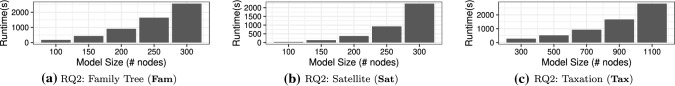
Model generation runtimes for large models

**Measurement setup:** We perform measurements in the ,  and  domains with increasing model sizes starting from 100 objects with a step size of 50/100 objects and timeout of 1 hour. We exclude the  domain from this experiment considering its low success rates for small models reported in **RQ1**. A single model is generated in each run. A campaign of 10 runs is executed for each measurement point and the median of *successful* execution times is taken (i.e., that provide a finite model as result within the given time). Additionally, we only gather measurement data for model sizes where 100% of runs are successful. In other words, we terminate the scalability measurements for a domain if any of the 10 runs at a particular size fails to output a finite model. For the  domain, we provide  instances as part of the initial model following the 1-to-20 ratio discussed in Sect. 6.2.

**Analysis of results:** Measurement results for **RQ2** are shown in Fig. 16. Interestingly, the proposed approach scaled best for the largest metamodel of the  case deriving models with 1100 objects within an hour. Furthermore, we were able to generate models with 1200 objects within the same time limit with a success rate of 80%. Model generation with 100% success rate scaled up to 300 objects for the  and  domains. However, root cause of scalability limits was very different (the numeric solver in  and graph solver in ). Interestingly,  turned out to be the most complex case study for assessing the use of numeric solvers.
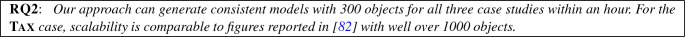


### RQ3: influence of exploration strategy

**Measurement setup:** We compare five state space exploration strategies:

- *Def* (used as a baseline) calls a numeric solver at every model generation step to repeatedly evaluate numeric constraints using the default strategy;
- *Qual* includes manually added qualitative abstractions of numeric constraints, which are assessed at every model generation step;
- *LowB* explicitly sets a lower bound of one for the number of newly created actors, which is the minimum requirement for  (this new actor will block the vision between the two actors included in the initial model);
- *Qual-LowB* incorporates the additional constraints used in *Qual* and in *LowB*;
- *Cust* uses the custom exploration strategy presented in Sect. 4.3 without additional qualitative abstractions or scope constraints.

The default exploration strategy *Cont* is used as our baseline. *Qual*, *LowB* and *Qual-LowB* also follow the default strategy with additional constraints which implicitly enforce particular decisions at each iteration. Due to the poor results reported in [67], we exclude measurements for a strategy that only makes numeric solver calls as a postprocessing step.

For **RQ3**, we perform measurements exclusively in the  domain, which has complex dependencies between structural and numeric constraints as well as complex numeric constraints. In fact, the complexity of the underlying numerical problem is shown in **RQ1** to pose a significant challenge when used with *Def*, which makes  an adequate case study for testing the influence of different exploration strategies. For the variations of the default strategy, we only use dReal as it is the more successful numeric solver which performed better for this domain (as shown in Sect. 6.2).

We aim to generate a single model that satisfies the constraints of . Ten runs are executed for each approach with a timeout of 5 minutes. Since the runtime of model generation is dominated by numeric solver calls (see **RQ1**), we calculate only the runtime of numeric solver calls and the success rates.

**Fig. 17:**
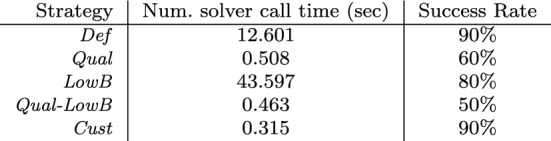
Numeric solver call time and success rate for exploration strategies

**Analysis of results:** Results are shown in Fig. 17. When using the default strategy without any additional hints (*Def*), we indicate a numeric solver call time of 12.601 s, with a 90% success rate. Adding qualitative abstractions of numeric constraints (*Qual* and *Qual-LowB*) does significantly reduce the numeric solver call time, as expected, given our result in [67], but also reduces the success rate. However, when qualitative abstractions are not included, we notice that when adding scope constraints (*LowB*) is detrimental to numeric solver call time. This is due to the heuristic used in the default strategy that is negatively affected by the additional scope constraint for this case study.

Despite achieving impressive runtime reductions by manually adding different constraints to the modeling domain, we notice that the most significant improvement is provided by the custom exploration strategy (*cust*). The latter reduces numeric solver call time by a factor of 40 without decreasing success rate.
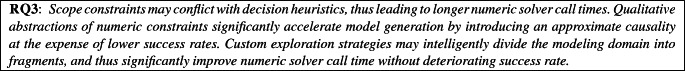


### RQ4: diversity

**Measurement setup:** To evaluate the structural diversity of the generated models, we used a neighborhood-based [57] internal diversity metric [70, 73], which correlates with mutation score in mutation testing scenarios. This metric calculates the proportion of different local neighborhoods of nodes included in a graph model. For this research question, we checked the structural diversity of models only.

We used a neighborhood range=4, which classifies two objects to be identical, if they cannot be distinguished with at most 4 links (hops). To measure structural diversity, the values of data objects are not taken into account (but data objects count as objects). We measured the diversity of 10$\times$10 models (we execute 10 runs, where each run produces 10 models) for case studies ,  and  with 100 objects, and measured the diversity of  with 18 objects. We compared the diversity of models generated with dReal and Z3.

**Fig. 18:**
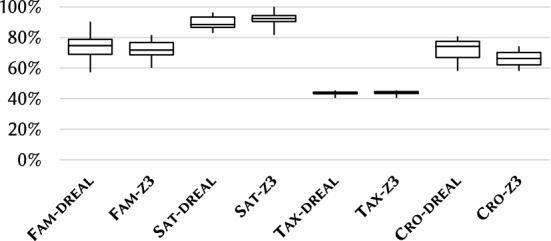
Internal diversity distributions


**Analysis of Results:** The distribution of internal diversity is illustrated in Fig. 18. The proportion of different object neighborhoods with respect to the number of objects is measured in percentage. , and  showed high internal diversity (between 65 and 80%), and  provided even higher diversity (around 90% median).  provided the lowest internal diversity (44%), which can be partially explained by the large number of similar attributes of the domain. Numeric solvers Z3 and dReal provided similar diversity.

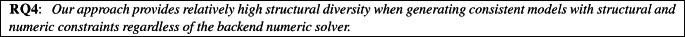


### Threats to validity

**Construct validity.** We have selected the  domain as a representative case study for the generation of critical traffic scenarios. In fact, it has been identified by Intel^5^ as a fundamental safety principle for autonomous vehicles. However, we do use various approximations (i.e., actors are modeled as rectangles, lanes have a fixed width) when implementing the case study to simplify the model generation task. We intend use the  domain as a proof of concept for generating critical traffic scenarios using our proposed approach.

We replicated the  case study [82] in a new technological context, which involved (1) to create an Ecore metamodel from an equivalent UML diagram and (2) to manually transform the OCL constraints into equivalent VQL graph patterns. The Ecore metamodel was kindly provided to us by the authors of [82], while we validated each replicated OCL constraint by performing manual equivalence checks. We used similar number of Household objects as in [82] and investigated the output models by graph visualization tools to ensure that similar model generation outputs are obtained, but we refrain from direct numerical comparison of execution times due to those technological differences.

**Internal Validity.** To strengthen internal validity, our experiments include a warm-up run executed prior to the actual measurements to decrease the fluctuation of runtimes caused by the Java VM instead of the natural fluctuation of solver runtimes. As the exploration strategy relies on some randomness, our scalability measurements only report cases with over 90% success rate—except for the  domain, where all success rates are reported explicitly.

**External Validity.** We mitigate threats to external validity by including a diverse set of case studies which involve calls to both a structural and a numeric solver. Furthermore, we incorporate and compare two distinct numeric solvers. We focused on numerical attributes as they are the most frequent data types. Handling models containing different kinds of attributes (e.g., string or bitvectors) can be a challenge in terms of performance (although Z3 does promise efficient background theorems for both [17, 89]). Additionally, the numeric values derived by the underlying numeric solver may not be diverse.

## Related work

We provide an overview of graph generation approaches that derive consistent graphs. We also discuss some key numerical abstractions and decision procedures, as well as traffic scenario generation approaches.

**Logic solver approaches.** These approaches translate graphs and WF constraints into a logic formulae and use underlying solvers to generate graphs that satisfy them. Back-end technologies used for this purpose include SMT solver such as Z3 [36, 66, 88], SAT-based model finders (like Alloy [35]) [3, 6, 13, 33, 40, 46, 49, 69, 74, 77, 78, 80], CSP-solvers [12, 14, 15, 28], theorem provers [5], first-order logic [8], constructive query containment [54], higher-order logic [30] and an incremental query engine [71].

For most of these approaches, scalability is limited to small models/counter-examples. These approaches are either a priori bounded (where the search space needs to be restricted explicitly) or they have decidability issues. Furthermore, handling of numeric constraints is not available for some of these approaches, particularly ones based on SAT-solvers and first-order logic formulations.

**Uncertain models.** Partial models are similar to uncertain models, which offer a rich specification language [18, 62] amenable to analysis. They provide a more intuitive, user-friendly language compared to 3-valued interpretations, but without handling additional WF constraints. Potential concrete models compliant with an uncertain model can be synthesized by the Alloy Analyzer [64], or refined by graph transformation rules [63].

**Strategies.** Iterative approaches generate models by multiple solver calls. An iterative approach is proposed specifically for allocation problems in [39] based on Formula. In [74], models are generated by calling Alloy in multiple steps, where each step extends the instance model by a few elements. Finally, an iterative, counterexample-guided synthesis is proposed for higher-order logic formulae in [47]. For these approaches, when scalability evaluation is included, it is limited to 50 nodes.

Some logic and numeric solvers provide an interface to configure the background theories and the strategy of the solving process. For example, the Z3 SMT solver [17] provides tactics and probes, and combinators to guide the solver. Similarly, portfolio solvers like [10] provide options to split reasoning tasks between a set of independent theorem provers.

**Symbolic model generation techniques.** Certain techniques use abstract (or symbolic) graphs for analysis purposes. A tableau-based reasoning method is proposed for graph properties [1, 52, 65], which automatically refines solutions based on WF constraints, and handles the state space in the form of a resolution tree as opposed to a partial model. When scalability evaluation is included, these techniques demonstrated to derive only small graphs ($<10$ objects).

Different approaches use abstract interpretation [57], or predicate abstraction [19, 29, 58] for partial modeling. In those approaches, concretization is used to materialize (typically small) counterexamples for designated safety properties in a graph transformation system. However, their focus is to support model checking of abstract graph transformation systems, which can evaluate complex trajectories, but do not scale in the size of the models.

**Hybrid approaches.** These approaches divide the model generation task into multiple sub-tasks and use a different underlying techniques to resolve each one. The PLEDGE model generation tool [82] provides such a scalable implementation by combining metaheuristic search for model structure generation with an SMT-solver based approach for attribute handling. The Evacon tool [34] implements a search-based evolutionary testing approach, followed by symbolic execution to generate tests for object-oriented programs. Autograph [66] sequentially combines a tableau-based approach for model structure generation with an SMT-solver-based approach for attribute handling. Such approaches combine multiple techniques in a sequential manner, which is a conceptual restriction for mutually dependent structural and numeric constraints. Moreover, none of these techniques assure completeness of model generation.

Another category of hybrid approaches involves assessing multiple components of the model generation task in parallel. This requires the implementation of a certain decision procedure such as DPLL(T) [21, 51] to iterate between underlying techniques, or combine them by sharing variables in their proofs [50]. Such decision procedures are presented alongside their associated properties (e.g., soundness and completeness) at an abstract level in [11, 51], which allows for formal reasoning about their implementations. However, those approaches handle graph-based models inefficiently [74, 87]; thus, the scalability of those techniques is limited.

**Numerical abstractions.** Handling numeric (integer or real) variables and constraints in model generation scenarios requires their abstract interpretation through numerical abstract domains [48, 79]. Numerical abstract domains may be used to summarize object attributes in value analysis of heap programs [19, 41, 45]. Summarized dimensions [29] were introduced to succinctly represent attributes of a potentially unbounded set of objects via multi-objects. This approach enables attribute handling in 3-valued partial models, and allows checking for refinements by abstract subsumption [2]. But these approaches do not generate graph models.

The uniqueness of our approach lies in combining numerical abstractions with partial models to guarantee soundness and completeness, while generating models with favorable scalability.

**Generating traffic scenarios.** Recently, testing autonomous vehicles with synthetic traffic scenarios has become a popular target for model generation. AsFault [20] proposes an approach using metaheuristic search and procedural content generation to derive challenging world maps for testing autonomous vehicles. This tool only generates static parts of a scenario: it provides no reasoning for dynamic components such as vehicles or pedestrians. A more complete scenario generation approach is proposed in [9], which uses a learnable evolutionary algorithm to guide exploration toward critical regions of the search space, and ultimately toward critical scenarios. Despite being able to generate both static and dynamic components, this approach lacks numeric reasoning, since numeric attributes are taken from a predefined finite set.

Other approaches use an underlying parametrized representation of scenarios. Paracosm [42] applies Halton sampling on the parameter space to generate scenarios according to coverage criteria. The approach proposed in [60] combines combinatorial interaction testing, backtracking and motion planning to generate test cases for regression testing of autonomous vehicles. The authors of [16] propose a weighted search-based approach to find test scenarios with *avoidable collisions*. In these cases, key information of the generated scenarios (e.g., the road map) must be provided as input, and only certain parameters (e.g., weather condition) are varied. This provides limited expressivity compared to our approach where we generate the entire underlying graph structure of the scenario from scratch.

## Conclusions

In this paper, we proposed an automated model generation approach to derive consistent models that satisfy structural and complex numeric constraints, which necessitates a bidirectional interaction between a graph solver and a numeric (SMT or quadratic) solver. As a conceptual novelty, we proposed refinement units that carry out consistency checking, decision, unit propagation and concretization steps in conceptual analogy with background theories used in SMT-solvers as part of an abstract DPLL procedure [51]. Therefore, refinement units can seamlessly incorporate different kinds of solvers (similarly to [50]) for handling attribute constraints in the presence of a graph solver that handles partial models. Additionally, the interactions between refinement units can be customized as domain-specific strategies. We implemented our approach in the Viatra Solver framework [68]. The source code of our approach is publicly available (https://github.com/viatra/VIATRA-Generator).

We carried out a detailed experimental evaluation of our approach in four complex case studies to assess scalability, diversity and the influence of custom strategies. We obtained favorable scalability results by consistently deriving models with over 250 objects in two cases within an hour, and models with over 1000 objects in a third case with same time limits. These model sizes are substantially larger than logic solver-based model generation approaches (e.g., Alloy or Z3) could derive in the presence of structural constraints (see [5, 71, 82]). In the fourth case study, which contains complex numeric constraints, we show the significant positive impact on runtime of custom exploration strategies. Moreover, our approach maintains other favorable quality attributes such as diversity and completeness investigated in depth in [70, 87].

## Footnotes

1 https://newsroom.intel.com/articles/rss-explained-five-rules-autonomous-vehicle-safety/#gs.tfv0wh.

2 A fragment here refers to a subset of domain components, such as metamodel classes or selected WF constraints unlike in [7] where it refers to a part of the search space.

3 https://github.com/ArenBabikian/publication-pages/wiki/Automated-Generation-of-Consistent-Models-Using-Qualitative-Abstractions-and-Exploration-Strategies.

4 12 $\times$ 2.2 GHz CPU, 64 GiB RAM, CentOS 7, Java 1.8, 12 GiB Heap.

5 https://newsroom.intel.com/articles/rss-explained-five-rules-autonomous-vehicle-safety/#gs.tfv0wh.
